# Predictors and clinical outcomes of incomplete hemostasis following transradial coronary intervention: the role of activated clotting time and procedural factors

**DOI:** 10.3389/fmed.2025.1661152

**Published:** 2025-12-16

**Authors:** Yujin Shin, Jae Myoung Lee, Ga-In Yu, Jae Seok Bae, Yun-Ho Cho, Jeong Yoon Jang, Yongwhi Park, Choong Hwan Kwak, Yong-Lee Kim, Min Gyu Kang, Kye-Hwan Kim, Jeong Rang Park, Hangyul Kim, Jong-Hwa Ahn

**Affiliations:** 1 Division of Cardiology, Department of Internal Medicine, Gyeongsang National University School of Medicine, Gyeongsang National University Changwon Hospital, Changwon, Republic of Korea; 2 Division of Cardiology, Department of Internal Medicine, Gyeongsang National University School of Medicine, Gyeongsang National University Hospital, Jinju, Republic of Korea

**Keywords:** transradial access, incomplete hemostasis, activated clotting time, percutaneous coronary intervention, radial ischemic complications, hemostasis devices, predictors

## Abstract

**Background:**

Major vascular complications are less frequent with trans-radial artery (TRA) access compared to transfemoral artery access. However, a substantial proportion of patients experience incomplete hemostasis following TRA intervention. This study aimed to identify factors associated with incomplete hemostasis and evaluate the predictive value of pre- and post-procedural activated clotting time (ACT).

**Methods:**

A total of 1,241 patients who underwent TRA intervention were included in a prospectively maintained single-center registry. Initial ACT was measured after sheath insertion, and final ACT was measured before sheath removal. Patients were categorized into complete and incomplete hemostasis groups based on achieving complete hemostasis within 2 h of continuous compression.

**Results:**

Incomplete hemostasis occurred in 230 patients (18%). Initial and final ACT values were significantly higher in the incomplete hemostasis group compared to the complete hemostasis group (initial ACT: 146 ± 37 s vs. 136 ± 32 s, *p* < 0.001; final ACT: 259 ± 85 s vs. 243 ± 72 s, *p* = 0.015). Multivariate analysis revealed that prolonged initial ACT (OR, 2.41; 95% CI, 1.71–3.39; *p* < 0.001) and final ACT (OR, 2.25; 95% CI, 1.52–3.30; *p* < 0.001) were independently significant predictors.

**Conclusion:**

Initial and final ACT measurements add predictive value to conventional risk factors for incomplete hemostasis in patients undergoing TRA intervention.

## Introduction

With continuous advances in catheter design, sheath technology, and patent hemostasis protocols, transradial artery (TRA) access has evolved into the preferred route for coronary interventions, offering a lower risk of vascular complications compared with transfemoral access (1, 2). The smaller caliber and superficial location of the radial artery allows for easier compressibility, making it advantageous in minimizing major access site bleeding. This approach has been supported by large-scale trials and meta-analyses demonstrating significant reductions in bleeding complications, mortality, and adverse cardiovascular events compared with transfemoral access (3, 4). In addition, recent improvements in device technology and standardized post-procedural hemostasis strategies have further optimized the safety and efficiency of TRA in contemporary practice (5).

Despite these advantages, a substantial subset of patients still experiences incomplete hemostasis after TRA interventions. This condition not only prolongs recovery time but also increases the risk of complications such as hematoma formation, pseudoaneurysm, and radial artery occlusion (RAO) (6, 7). Identifying patients at risk of incomplete hemostasis is critical for improving outcomes and optimizing procedural planning.

Numerous factors influence hemostasis after TRA intervention, including patient-specific characteristics (e.g., age, sex, and vascular health), procedural variables (e.g., sheath size and anticoagulation regimen), and post-procedure management (2, 6, 7). Among these, activated clotting time (ACT), a readily measurable indicator of anticoagulation status, has been widely used during the percutaneous coronary intervention (PCI) to monitor and guide heparin therapy (8, 9). However, its role in predicting hemostasis outcomes remains underexplored.

In this study, we aimed to investigate the association between pre- and post-procedural ACT values and incomplete hemostasis in patients undergoing TRA coronary intervention. By examining clinical, procedural, and laboratory data from a large single-center registry, we sought to identify predictors of incomplete hemostasis and evaluate the utility of ACT measurements in risk stratification. These findings could inform clinical strategies to enhance hemostasis success rates and reduce associated complications in patients undergoing TRA procedures.

## Methods

### Study population

**Figure 1:**
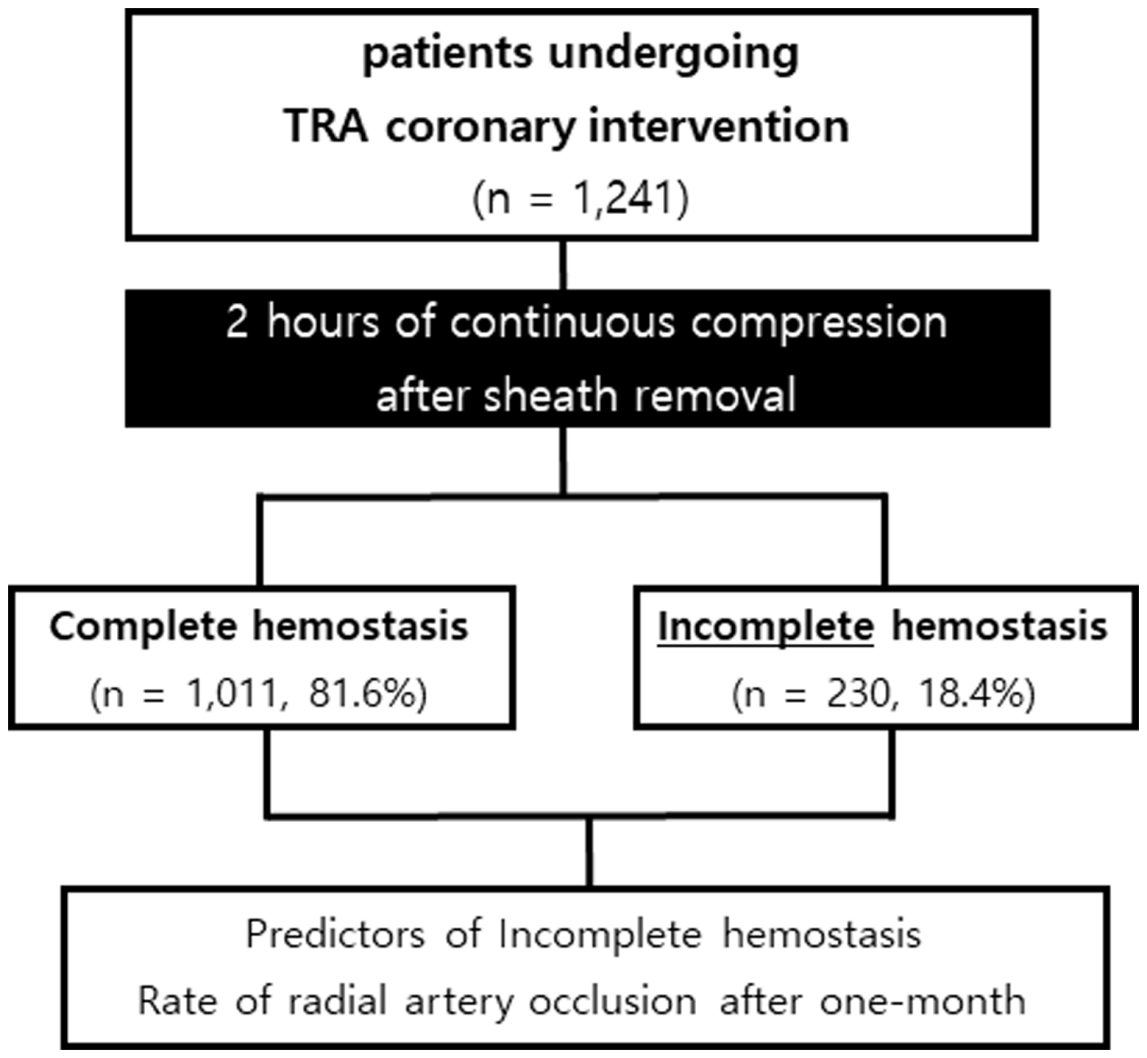
Profile of the patient. TRA, transradial artery.

Between March 2020 and February 2024, a total of 1,241 consecutive patients undergoing transradial coronary intervention were included in a prospectively maintained single-center registry, which aimed to evaluate the achievement rate of successful hemostasis according to ACT values measured immediately after sheath insertion (initial ACT) and before sheath removal (final ACT). Clinical, laboratory, and outcome data were collected by a trained study coordinator using a standardized case report form and protocol. The institutional review board of CGNUH approved this study. All subjects gave written informed consent before performing this investigation. Patients were categorized as a complete hemostasis group or an incomplete hemostasis group according to the achievement of complete hemostasis at 2 h after continuous compression at the puncture site. The flow of patient selection for this study is shown in Figure 1.

### Transradial access and measurements of initial ACT

After radial artery puncture, a hydrophilic-coated introducer sheath catheter was placed for coronary catheter insertion. Initial ACT samples were drawn through the arterial sheath immediately after sheath catheter placement. To clear the sample from the flush solution contaminated by heparin, 10 mL of blood was withdrawn before taking 4 mL of the ACT sample. ACT was measured using the Hemochron Jr. Signature+ (International Technidyne Corporation). Shortly after taking a blood sample, unfractionated heparin of 50 IU/kg was administered. When percutaneous coronary intervention (PCI) was needed, additional heparin of 50 IU/kg was administered through the sheath before introducing the guided catheter. Additional boluses were then added intravenously according to the physician’s choice.

### Hemostasis and measurements of final ACT

After the completion of the procedure, final ACT samples were drawn through the arterial sheath immediately before removal. The standard method of hemostasis included applying a gauze pad compression for at least 2 h, with additional hours of compression when needed. Patients received radial artery compression by a gauze pad folded into a cuboid shape, fastened with 3MTM DuraporeTM surgical tape. The gauze pad was not impregnated with prothrombotic material. The hemostasis device was removed as soon as possible once complete hemostasis was achieved. The first attempt to remove the hemostasis device was performed 2 h after the procedure and then every hour.

### Assessment of RAO

When the gauze pad or the device was removed, we applied a simple dressing to protect the puncture wound. Patients were monitored with caution after coronary angiography. They were evaluated at 1 month by investigators to evaluate RAO by palpation of pulse and/or Doppler ultrasound of the radial artery.

### Statistical analysis

Patients were divided into two subgroups: complete and incomplete hemostasis groups. Data were displayed separately for each group. Continuous variables are expressed as means ± standard deviations. Categorical variables are presented as absolute numbers and proportions (%). Overall comparisons between groups were performed using Student’s *t*-test for continuous variables and the chi-squared test or Fisher’s exact test when the Cochran rule was not met for categorical variables. A receiver-operating characteristic (ROC) curve analysis of the initial and final ACTs was performed so that the optimal cutoff value for incomplete hemostasis could be determined. The optimal cutoff point of the ACT was calculated using the Youden method, which defined the cutoff in terms of the maximum sum of sensitivity and specificity. The predictors of incomplete hemostasis were evaluated using logistic regression with the following candidate variables: age, sheath size, final systolic blood pressure, pretreatment with P2Y12 inhibitors, ultrasound-guided access, initial ACT, and final ACT. To minimize the potential impact of missing data in the regression analysis, the following steps were performed: missing continuous variables were imputed using their sample means (no missing data were present for categorical variables); backward variable selection (stay criterion, *p* < 0.10) was applied to the imputed dataset to identify significant predictors; and a final multivariable logistic regression model including only the selected covariates was re-estimated using complete-case data. The overall proportion of missing data was low (<3% across all variables). Mean imputation was used only for descriptive purposes, and all multivariable analyses were conducted on complete cases. A *p-value of* < 0.05 indicated a statistically significant difference. All statistical analyses were conducted with IBM/SPSS (21.0 for Windows, IBM/SPSS, Chicago, IL, United States).

## Results

A total of 1,241 patients who underwent TRA coronary intervention were classified into the complete hemostasis group (1,011 patients, 81.6%) or the incomplete hemostasis group (230 patients, 18.4%) according to complete hemostasis at 2 h after continuous compression at the puncture site.

### Baseline characteristics of the incomplete hemostasis group after TRA coronary intervention

Regarding baseline clinical characteristics, there was no significant difference in age, sex, procedure time, or initial blood pressure between the two groups. However, patients with incomplete hemostasis had a significantly higher final diastolic blood pressure, more patients were treated with large-sized radial sheaths, fewer patients used ultrasound guidance for radial access, and more patients received pretreatment of P2Y12 inhibitors than those with complete hemostasis (Table 1). The baseline characteristics of the two groups are summarized in Table 1.

**Table 1:** Characteristics of the study population.

Characteristics of the study population	Complete hemostasis	Incomplete hemostasis	*P*
	(*N* = 1,011)	(*N* = 230)	
Male	316 (31.3%)	82 (35.7%)	0.226
Age (years)	61.5 ± 12.1	63.0 ± 12.2	0.095
Sheath size			**0.021**
5Fr	251 (24.8%)	40 (17.4%)	
6Fr	760 (75.2%)	190 (82.6%)	
Systolic BP (initial, mmHg)	116.6 ± 19.2	113.2 ± 18.0	0.084
Diastolic BP (initial, mmHg)	76.1 ± 14.6	75.5 ± 13.6	0.622
Systolic BP (final, mmHg)	117.9 ± 18.2	119.7 ± 17.6	0.164
Diastolic BP (final, mmHg)	74.1 ± 14.1	77.0 ± 13.7	**0.005**
Procedure time (min)	36.9 ± 24.5	39.3 ± 22.9	0.162
Pretreatment of P2Y12 inhibitors	434 (42.9%)	146 (63.5%)	**<0.001**
Use of potent P2Y12 inhibitors	182 (18.0%)	42 (18.3%)	0.99
Coronary angiography only	305 (30.2%)	81 (35.2%)	0.157
Ultrasound guidance access	824 (81.5%)	170 (73.9%)	**0.012**

Bold values indicate statistical significance (*p* < 0.05).

### Predictive value of pre- and post-ACT in incomplete hemostasis after TRA coronary intervention

There were no significant differences in laboratory parameters except the initial and final ACT levels. The mean value of initial ACT was significantly higher in patients with incomplete hemostasis than in those with complete hemostasis (146 ± 37 s vs. 136 ± 32 s, *p* < 0.001). Final ACT was also significantly higher in patients with incomplete hemostasis than in those with complete hemostasis (259 ± 85 s vs. 243 ± 72 s, *p* = 0.015). Laboratory parameters for the two groups are summarized in Table 2.

**Table 2:** Laboratory parameters of the study population.

	Complete hemostasis	Incomplete hemostasis	*P*
	(*N* = 1,011)	(*N* = 230)	
Hemoglobin (g/dL)	13.9 ± 5.2	13.7 ± 1.7	0.33
Platelets (10^9^/L)	239.6 ± 70.7	237.2 ± 63.5	0.71
Total cholesterol (mg/dL)	177.9 ± 46.1	179.6 ± 47.4	0.71
Fibrinogen (g/L)	320.6 ± 85.1	310.4 ± 73.8	0.24
Albumin (g/dL)	4.4 ± 2.1	4.3 ± 0.4	0.23
NT-proBNP (pg/mL)	815 ± 3419	830 ± 3129	0.97
activated clotting time (initial)	136 ± 32	146 ± 37	**<0.001**
activated clotting time (final)	243 ± 72	259 ± 85	**0.007**

Bold values indicate statistical significance (*p* < 0.05).

**Figure 2:**
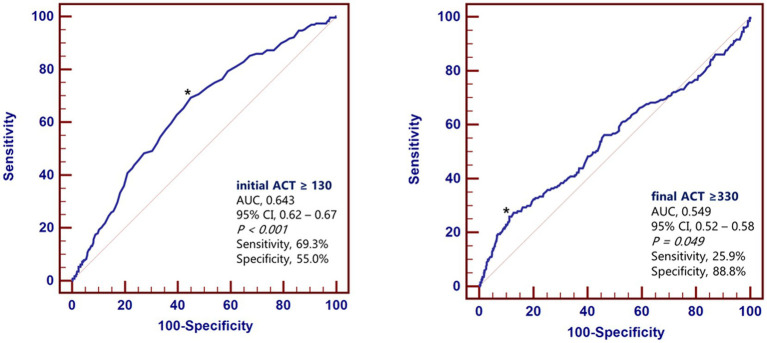
Predictive value of activated clotting time for a prolonged time to hemostasis. ACT, activated clotting time; AUC, area under the curve.

In the ROC curve analysis of initial and final ACTs predicting postprocedural incomplete hemostasis, AUCs were 0.64 (95% CI, 0.62–0.67, *p* < 0.001) and 0.55 (95% CI, 0.52–0.58, *p* < 0.001), respectively. Optimal initial and final ACT cutoff values for postprocedural incomplete hemostasis were 130 s (sensitivity, 69%; specificity, 55%; PPV, 26%; NPV, 89%) and 330 s (sensitivity, 26%; specificity, 89%; PPV, 34%; NPV, 84%), respectively (Figure 2). OR for postprocedural incomplete hemostasis was 2.61 (95% CI, 1.91–3.56, *p* < 0.001) for those with prolonged initial ACT (≥130 s) and 2.56 (95% CI, 1.75–3.73, *p* < 0.001) for those with prolonged final ACT (≥330 s) (Figure 2 and Table 3).

**Table 3:** Univariate and multivariate predictors of incomplete hemostasis.

	Univariate analysis		Multiple logistic regression analysis	
	OR (95% CI)	*P*	OR (95% CI)	*P*
Age ≥60 years old	1.46 (1.09–1.96)	*0.012*	*–*	*–*
Sheath size (6Fr vs. 5Fr)	1.60 (1.11–2.32)	*0.012*	*–*	*–*
Final systolic BP ≥ 140 mmHg	1.66 (1.04–2.66)	*0.035*	*–*	*–*
Pretreatment of P2Y12 inhibitors	2.35 (1.56–3.56)	*<0.001*	2.08 (1.50–2.89)	*<0.001*
Ultrasound guidance access	0.61 (0.43–0.86)	*0.011*	*-*	*-*
Initial ACT ≥130 s	2.56 (1.75–3.73)	*<0.001*	2.41 (1.71–3.39)	*<0.001*
Final ACT ≥330 s	1.60 (1.11–2.32)	*<0.001*	2.25 (1.52–3.30)	*<0.001*

### Predictors of incomplete hemostasis after TRA coronary intervention

**Figure 3:**
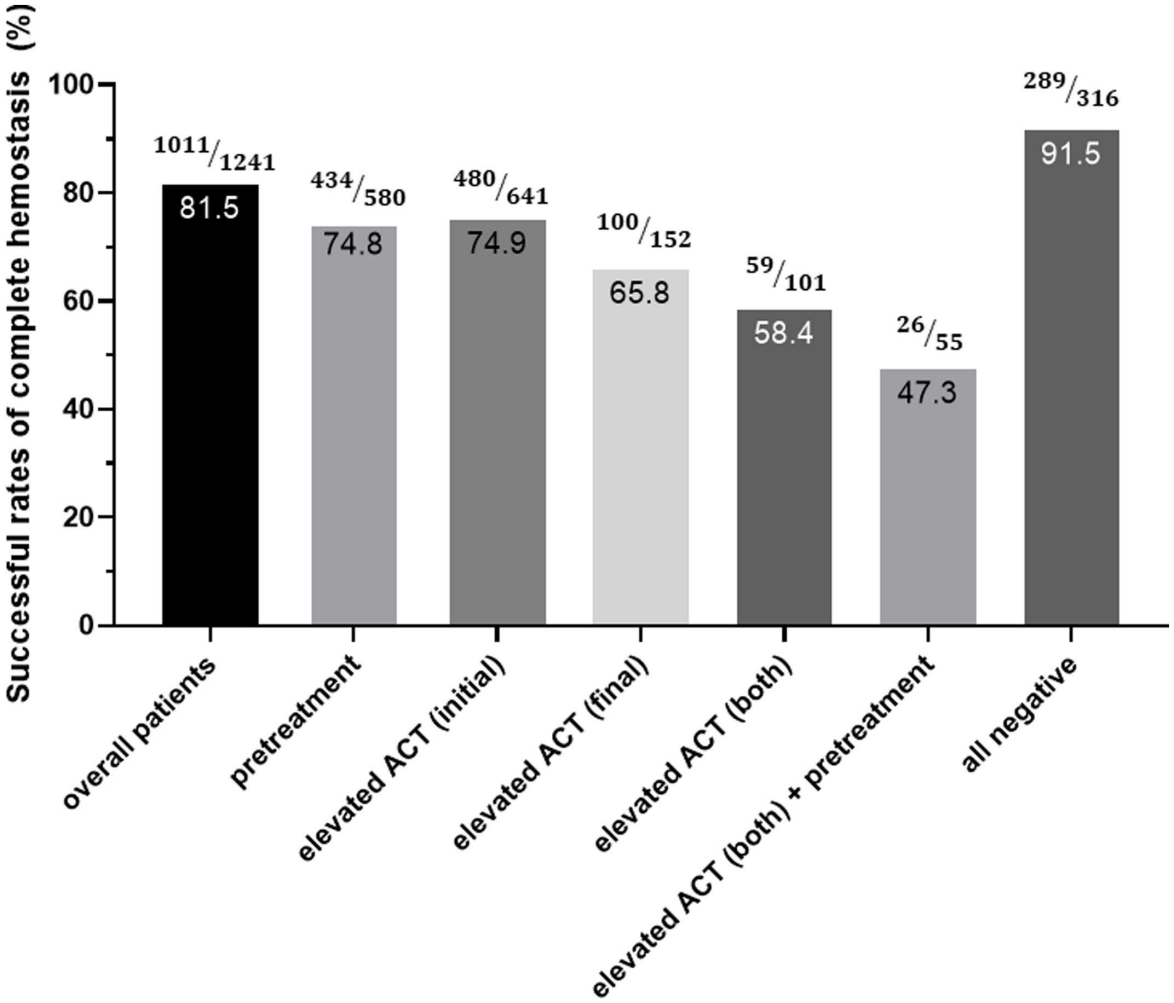
Successful rates of complete hemostasis after 2 h of continuous compression. ACT, activated clotting time.

Logistic regression analysis results for predicting incomplete hemostasis are presented in Table 3. In unadjusted analyses, older age, large sheath size, pretreatment with P2Y12 inhibitors, use of ultrasound guidance, and prolonged ACT values (initial ACT ≥130 s and final ACT ≥330 s) were significantly associated with incomplete hemostasis. However, after adjusting for covariates, only prolonged initial ACT (OR 2.41, 95% CI 1.71–3.39, *p* < 0.001) and prolonged final ACT (OR 2.25, 95% CI 1.52–3.30, *p* < 0.001) remained independently significant predictors. Notably, pretreatment with P2Y12 inhibitors remained an independent predictor of incomplete hemostasis after multivariable adjustment (OR 2.08, 95% CI 1.50–2.89; *p* < 0.001), indicating a consistent association between enhanced platelet inhibition and higher risk of incomplete hemostasis. Finally, we stratified the success rates of hemostasis after 2 h of continuous compression according to pretreatment with P2Y12 inhibitors and prolonged ACT values. The overall success rate of hemostasis was 81.5%. This rate was lower among patients with P2Y12 pretreatment or prolonged ACT levels (74.8 and 47.3%, respectively) (Figure 3).

### Assessment of RAO

**Figure 4:**
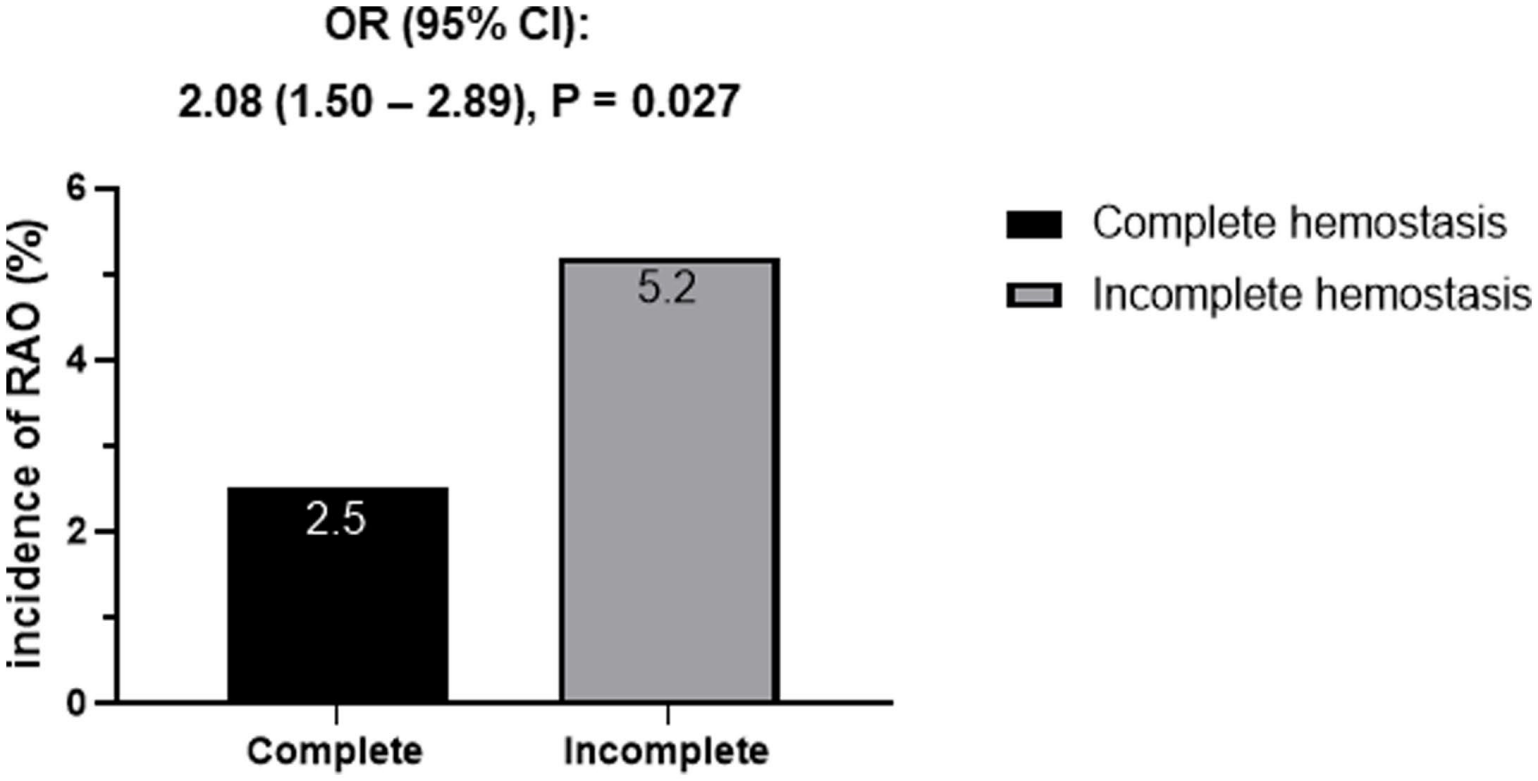
Incidence of RAO according to the hemostasis status.

The incidence of radial artery occlusion (RAO) according to hemostasis status is presented in Figure 4. Overall, 37 patients (3.0%) developed RAO after TRA coronary intervention. Patients with incomplete hemostasis had a significantly higher incidence of RAO than those with complete hemostasis (5.2% vs. 2.5%, *p* = 0.027). In logistic regression analysis, incomplete hemostasis was independently associated with the occurrence of RAO (OR 2.08, 95% CI 1.50–2.89; *p* = 0.027) (Figure 4).

## Discussion

This study provides significant insights into the predictors and clinical outcomes of incomplete hemostasis following trans-radial artery (TRA) coronary intervention. Several key findings are worth highlighting and contextualizing in light of the existing literature and clinical practice.

First, incomplete hemostasis was observed in 18.4% of patients, a non-negligible proportion, underscoring the importance of identifying reliable predictors for this complication. Among baseline characteristics, higher final diastolic blood pressure, use of larger radial sheaths, and pretreatment with P2Y12 inhibitors were associated with incomplete hemostasis. These findings align with previous studies demonstrating the impact of patient hemodynamics and procedural factors on vascular outcomes (7, 10, 11). Second, both initial and final activated clotting time (ACT) emerged as significant predictors of incomplete hemostasis. Prolonged initial ACT (≥130 s) and final ACT (≥330 s) were independently associated with a higher risk of incomplete hemostasis, as shown by logistic regression analysis. The ROC analysis confirmed the predictive value of these parameters with reasonable sensitivity and specificity. Although the AUC values for both initial and final ACT were modest, suggesting limited discriminative performance, the primary aim of this study was to identify ACT-related trends rather than to establish a predictive model. Accordingly, calibration and decision-curve analyses were not performed, as the study was not intended for model development. ACT may thus serve as a supportive indicator to assist in individualized hemostasis management rather than a stand-alone screening tool. These findings highlight the utility of ACT as a practical, point-of-care tool for identifying patients at elevated risk during TRA intervention (8, 9). This expands its role beyond monitoring anticoagulation levels to stratifying hemostasis risk. Third, the occurrence of incomplete hemostasis was correlated with a higher incidence of radial artery occlusion (RAO), observed in 5.2% of patients compared to 2.5% in the complete hemostasis group. This reinforces the critical role of achieving complete hemostasis not only to minimize immediate bleeding complications but also to prevent long-term vascular sequelae such as RAO, which can limit future trans-radial access for coronary or neuroendovascular procedures (12). Laser Doppler perfusion imaging (LDPI) has recently been applied to quantify hand perfusion and microcirculatory flow after transradial procedures. Compared with standard Doppler assessment, LDPI provides a more detailed and quantitative analysis of tissue perfusion, allowing for an improved evaluation of radial artery patency and collateral circulation. Although LDPI was not used in the present study, future investigations incorporating this technique may offer additional insight into the physiological consequences of RAO (13, 14).

The stratification of hemostasis success rates based on ACT values and pretreatment with P2Y12 inhibitors revealed a synergistic effect of these factors. Patients with both prolonged ACT and P2Y12 inhibitor pretreatment exhibited substantially lower success rates for hemostasis, emphasizing the need for individualized procedural strategies in such populations (15, 16).

### Clinical implications

The findings from this study support the integration of ACT measurements into the routine peri-procedural assessment of TRA coronary interventions. By identifying patients with prolonged ACT values, clinicians can adopt tailored strategies to optimize hemostasis, such as modifying compression duration or using advanced hemostasis devices. Furthermore, careful consideration of anticoagulation regimens, particularly P2Y12 inhibitor pretreatment, is warranted to balance thrombotic and bleeding risks.

### Limitations

This study has several limitations. Being a single-center observational study, the findings may not be generalizable to all centers using varied hemostasis techniques. Second, since hemostasis in this study was achieved using a gauze pad secured with adhesive tape rather than a contemporary patent-hemostasis device (e.g., TR-Band), the generalizability of our results to current practice may be limited. However, as a single, uniform compression technique was consistently applied to all patients, internal validity was preserved, allowing reliable comparison of ACT-related differences. Third, because additional heparin dosing, P2Y12 pretreatment regimens, and ultrasound guidance were not fully standardized and were left to operator discretion, residual confounding cannot be excluded. However, all patients received an initial bolus of 50 IU/kg of unfractionated heparin and underwent systematic ACT monitoring, ensuring a consistent baseline anticoagulation strategy.

Additionally, while ACT was demonstrated as a robust predictor, its threshold values may require further validation in larger, multicenter cohorts. Finally, the study did not explore other potential biomarkers or imaging modalities that could complement ACT measurements in predicting hemostasis outcomes.

## Conclusion

In summary, prolonged initial and final ACT values are independently significant predictors of incomplete hemostasis following TRA coronary intervention. These findings underscore the value of ACT as a simple, actionable tool for risk stratification and procedural planning. Efforts to optimize hemostasis, particularly in high-risk patients, are essential to reduce the incidence of complications such as RAO and enhance procedural outcomes. Future research should focus on validating these findings across diverse populations and exploring adjunctive strategies to improve hemostasis success rates.

## Data Availability

The raw data supporting the conclusions of this article will be made available by the authors, without undue reservation.
